# Immunoadjuvant Nanoparticles as Trojan Horses for Enhanced Photo-Immunotherapy in the Treatment of Triple-Negative Breast Cancer

**DOI:** 10.3389/fphar.2022.883428

**Published:** 2022-05-05

**Authors:** Jinxian Wu, Shanyong Wang, Shanshan Liu, Fang Liu, Feifan Zhou

**Affiliations:** ^1^ Key Laboratory of Biomedical Engineering of Hainan Province, School of Biomedical Engineering, Hainan University, Haikou, China; ^2^ One Health Institute, Hainan University, Haikou, China

**Keywords:** triple-negative breast cancer, nanoparticles, phototherapy, immunotherapy, metastasis, immune response

## Abstract

Treatment of triple-negative breast cancer (TNBC) faces great challenges due to high invasiveness and poor prognosis. Therefore, effective treatment methods are urgently needed to control primary tumors and suppress distant tumors. Herein, we employed glycated chitosan (GC), a polysaccharide macromolecular immunoadjuvant, to construct a self-assembly GC@ICG nanoparticle which is accessible to tumor cells for synergistic cancer treatment based on the combination of phototherapy and immunotherapy. In this strategy, the self-associated synthesis of spherical GC@ICG significantly improved the stability of ICG and endowed GC with Trojan Horses in tumor cells to enhance tumor immunogenicity. A bilateral 4T1 tumor-bearing mouse model was established to evaluate the therapeutic outcomes and specific host antitumor immune response. Finally, GC@ICG-based phototherapy can directly eliminate primary tumors and resist the progression of untreated distant tumors. In addition, compared to the treatment of L + GC + ICG, GC@ICG-based phototherapy was evidenced to suppress lung metastasis and enhance infiltration of CD8^+^ T cells in untreated distant tumors. Therefore, this design shows promise in addressing the challenges of the treatment of TNBC.

## Introduction

Breast cancer (BC) is the most common malignant cancer among women. It is estimated that female breast cancer has overtaken lung cancer for the first time as the most commonly diagnosed cancer globally, accounting for 11.7% of the new cases in 2020 (Sung et al., 2021). The 5-year relative survival rate of female patients with localized breast tumors was 99%, whereas it was only 28% for those with distant breast tumors (Siegel et al., 2021). Triple-negative breast cancer (TNBC) is a highly heterogeneous subtype of breast cancer, and the median overall survival of patients with metastatic tumors was approximately 1 year (Waks and Winer, 2019). TNBC was characterized by the lack of three receptors, including estrogen receptor (ER), progesterone receptor (PR), and human epidermal growth factor 2 (Her 2) (Garrido-Castro et al., 2019; Hwang et al., 2019). In clinic, chemotherapy followed by surgery has been the mainstream therapeutic strategy for the treatment of TNBC, while its inherent rapid metastasis and early recurrence could result in an unsatisfactory prognosis (Poggio et al., 2018; Yin et al., 2020). Recent progress has been made in immunotherapy for a variety of malignancies, such as leukemia, melanoma, and bladder cancer, while the poor immunogenicity presented by TNBC would develop immune surveillance escape and lead to undesirable therapeutic outcomes (Hucks and Rheingold, 2019; Weiss et al., 2019; van Puffelen et al., 2020; Wang et al., 2020). Therefore, it is imperative to improve the host immune response and promote the elimination of both primary and distant tumors.

Photo-immunotherapy (PIT), a novel oncological treatment that combines phototherapy and immunotherapy, can achieve a synergistic photothermal immune effect and induce a specific antitumor immune response to eliminate the primary tumors and prevent distant tumors (Zhou B et al., 2018; Wang et al., 2019; Wang et al., 2021; Liu et al., 2022). It is reported that mono-photothermal therapy enables inducing immunogenic cell death by raising the temperature of targeted tumors to an appropriate range and facilitating the release of damage-associated molecular patterns (DAMPs) (Sato et al., 2018; Huang et al., 2021). However, the host immunity induced by mono-phototherapy is inadequate to provide an effective control of distant metastases (Gordon et al., 2017; Nam et al., 2018; Hu et al., 2021). In recent years, immunoadjuvant has attracted much attention due to the ability of immune response enhancement (Chen et al., 2016; Ng et al., 2018). In a clinical trial of PIT for advanced breast cancer patients, glycated chitosan (GC) was introduced as an immunoadjuvant to amplify the immune response induced by indocyanine green (ICG)-based phototherapy. Notably, among the eight patients available for PIT evaluation, complete response was achieved in one patient, partial response was achieved in four patients, stable disease was achieved in one patient, and progressive disease was observed in two patients. Furthermore, lung, lymph node, and liver metastases were significantly decreased in several patients (Li et al., 2011). Although ICG is an FDA-approved near-infrared (NIR) photosensitizer for clinical uses, the aggregation at the high concentration and rapid clearance rate limit the further application of ICG (Chen et al., 2013; Wang et al., 2018). Moreover, the high molecular weight makes GC, a polysaccharide formed by attaching galactose molecules to the chitosan molecules, only disperse slowly extracellularly (Zhou et al., 2012). Thus, allowing drugs to enter cells more efficiently and making the residence time of ICG in tumors longer show the prospect of obtaining a better photothermal effect and tumor immune response.

**SCHEME 1:**
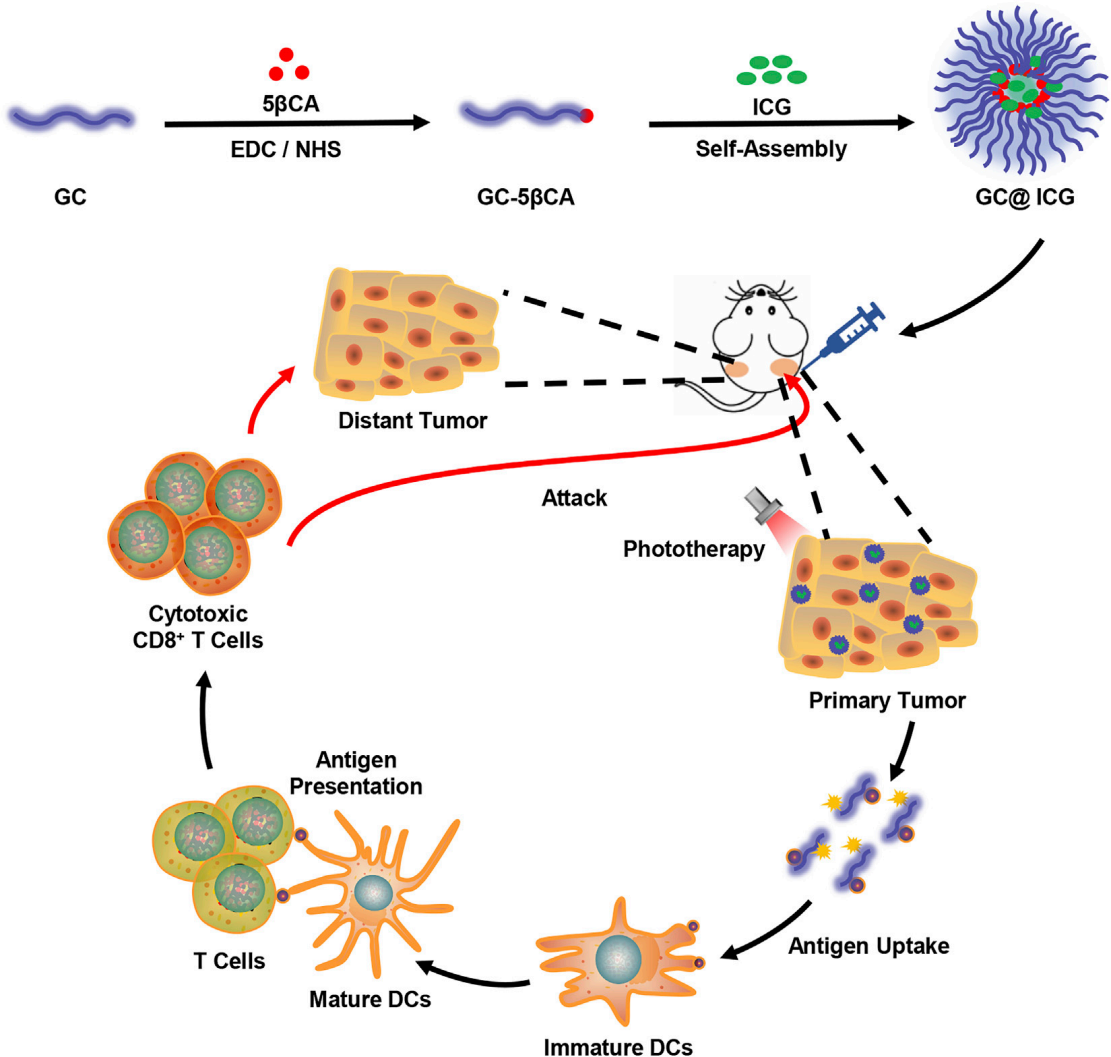
Schematic illustration showing GC@ICG-based photo-immunotherapy for triple-negative breast cancer.

Herein, amphiphilic 5β-cholanic acid-modified GC (GC-5βCA) and ICG were assembled to form GC@ICG nanoparticles for synergistic treatment of TNBC. Specifically, GC@ICG particles could ideally mimic the Trojan Horses to deliver GC and ICG into tumor cells to ablate primary tumors. Furthermore, a specific antitumor immune response was induced and amplified to suppress the distant tumors, resulting in an excellent antitumor effect (Scheme 1). In this study, the antitumor effect and immune response were investigated *in vitro* and *in vivo.*

## Materials and Methods

### Preparation of GC@ICG

In order to synthesize GC@ICG nanoparticles, 5βCA (8 mg) was dissolved in methanol, followed by 40 µl of EDC and NHS. The mixture reacted at ambient temperature for 30 min (pH 6–6.5). Then, GC (1.3 ml 1% GC dispersed in 50% methanol and 50% deionized water) was added to the polymer solution (pH 7–7.5). After stirring at an ambient temperature condition for 24 h, the resulting solution dialyzed (molecular weight cut-off = 50 kDa) for 72 h to remove nonbinding 5βCA and excess EDC/NHS. Next, ICG (1 mg/ml, 700 µl) was administered in the prepared solution, and the solution was sonicated three times using an ultrasound apparatus (JY88-IIN, Scientz, China) for 2 min each. Finally, GC@ICG was centrifuged for 20 min to remove excess ICG, and the precipitate was re-suspended in deionized water for further use.

### Characterization of GC@ICG

For FTIR spectra acquisition, the freeze-dried samples were mixed with potassium bromide powder (KBr) and pressed into a thin film, and then a Fourier transform infrared spectroscopy (FTIR, Frontier, PerkinElmer, US) was used to collect FTIR spectra of our samples. The morphology and size of the GC@ICG were examined using SEM (S-3000N, Hitachi, Japan). Dynamic light scattering (Zetasiaer Nano ZSE, Malvern, United Kingdom) was used to measure the average diameter and size distribution of nanoparticles. UV-vis absorption spectra were detected by a UV-visible spectrometer (Evolution 220, Thermo Fisher, United States). The photothermal images were performed by an infrared thermal camera (226S, FOTRIC, Shanghai, China).

### Cell Culture

4T1 cells, murine mammary tumor cells, were cultured in RPMI-1640 (Gibco) medium containing 10% fetal bovine serum and 1% penicillin and streptomycin at 37 °C under 5% CO_2_.

In order to collect bone marrow dendritic cells (DCs), the femurs and tibiae of BALB/C female mice were collected, and surrounding muscle tissues were thrown away. RPMI-1640 medium without FBS was used to wash the bone marrow to prepare cell suspension. Then, 10% FBS was added to the medium and incubated for 2 h under 5% CO_2_. Subsequently, the supernatant was removed, and RPMI-1640 medium (10% FBS, 20 ng/mL GM-CSF (PeproTech, NJ, United States)) was re-added for further culture.

### Determination of Cell Cytotoxicity

The CCK-8 (Dojindo, Kumamoto, Japan) assay was used to assess the cytotoxic effect of GC@ICG NPs. Briefly, 4T1 cells were placed into 96-pore plates and incubated for 10 h (37°C, 5% CO_2_), followed by the addition of GC@ICG at different concentrations (ICG 0.3, 1, and 3 μg/ml; corresponding GC 10, 33.3, and 100 μg/ml, respectively) and irradiated with or without an 808-nm laser at 0.8 W/cm^2^ for 2 min, respectively. Cells were washed three times with buffer after 24 h and incubated with 10 µL CCK-8 at 37 °C for 2 h. Then, the absorbance at 450 nm was measured by a microplate reader (AMR-100, Allsheng, Hangzhou, China).

For subcellular localization analysis, 4T1 cells were cultured in a glass-bottom cell culture dish and incubated at 37 °C for 12 h. Then, cells were incubated with GC@ICG-FITC for 6 h and then stained with LysoTracker Red (lysosome indicator, Invitrogen, NY, United States) and Hoechst 33342 (nucleus indicator, Invitrogen) according to the supplier’s instructions. Images were taken with a confocal laser scanning microscope (FV3000, OLYMPUS, Japan).

For ROS generation analysis, 4T1 cells were seeded in a 24-well plate at a density of 5×10^5^ cells for each well, and GC@ICG was incubated with the cells for 12 h. After irradiation by the 808-nm laser (0.8 W/cm^2^) for 5 min, the ROS in 4T1 cells were stained with 2′,7′-dichlorofluorescin diacetate (DCFH-DA, Invitrogen, United States), and the fluorescence intensity was analyzed by fluorescence microscope (MD43-N, Mshot, Guangzhou, China).

For cell death analysis, 4T1 cells were placed into 24-well plates and cultured at 37°C for 10 h. Then, 4T1 cells were co-cultured with or without GC@ICG for 12 h, followed with or without 808 nm laser irradiation (0.8 W/cm^2^ for 5 min). Then, calcein acetoxymethyl ester (calcein-AM, Invitrogen)/propidium iodide (PI, 5 μg/ml, Sigma-Aldrich) was added 1 hour after laser irradiation and then detected by fluorescence microscope.

For evaluation of DC maturation, DCs were incubated with tumor cells for 12 h. Then, the treated cells were stained with anti-mouse PE-CD86 and FITC-CD11c (BioLegend, United States) and detected by flow cytometry (CytoFLEX, Beckman Coulter, CA, United States).

### Establishment of Animal Model and *In Vivo* Treatment

BALB/C female mice at 6–8 weeks were obtained from the Guangdong Medical Laboratory Animal Center. Animal handling procedures follow the guidelines of the Regional Ethics Committee for Animal Experiments. In order to establish a bilateral breast tumor model, 1.2 × 10^5^ and 0.8 × 10^5^ 4T1 cells were subcutaneously implanted into the left and right breast areas of the mice, respectively. When the primary tumor volume reached 200 mm^3^, the mice were assigned into five groups randomly (*n* = 3), including control, L, GC@ICG, L + GC + ICG, and L + GC@ICG groups. In order to study the photothermal effect of GC@ICG, the mice in the L + GC + ICG and L + GC@ICG groups were injected with 100 µL of the mixture of GC and ICG, and GC@ICG (GC 1%, ICG 20 μg/ml), respectively. Then, the mice were anesthetized, and primary tumors were irradiated with an 808-nm laser (0.8 W/cm^2^). The temperature of the tumor surface was recorded by an infrared thermal imaging camera. The tumor volumes and body weights of the mice were recorded for 7 days.

### Immunofluorescence Assay

Tumors were collected and fixed with 4% paraformaldehyde and cut into 5 μm thick sections. FITC anti-mouse CD8a antibody, PE anti-mouse CD25, and FITC anti-mouse CD206 (MMR) antibody (BioLegend) were used to stain samples. Then, they were analyzed by fluorescence microscopy.

### Statistical Analysis

All measurements were performed in biological triplicate, and the data are presented as mean ± SD. ANOVA was used for evaluation. The *p*-value < 0.05 was determined as significant and all significant values were performed as follows: *∗p* < 0.05, *∗∗p* < 0.01, and ^*#*^ *p* < 0.05.

## Results and Discussion

### Preparation and Characterization of GC@ICG

**FIGURE 1:**
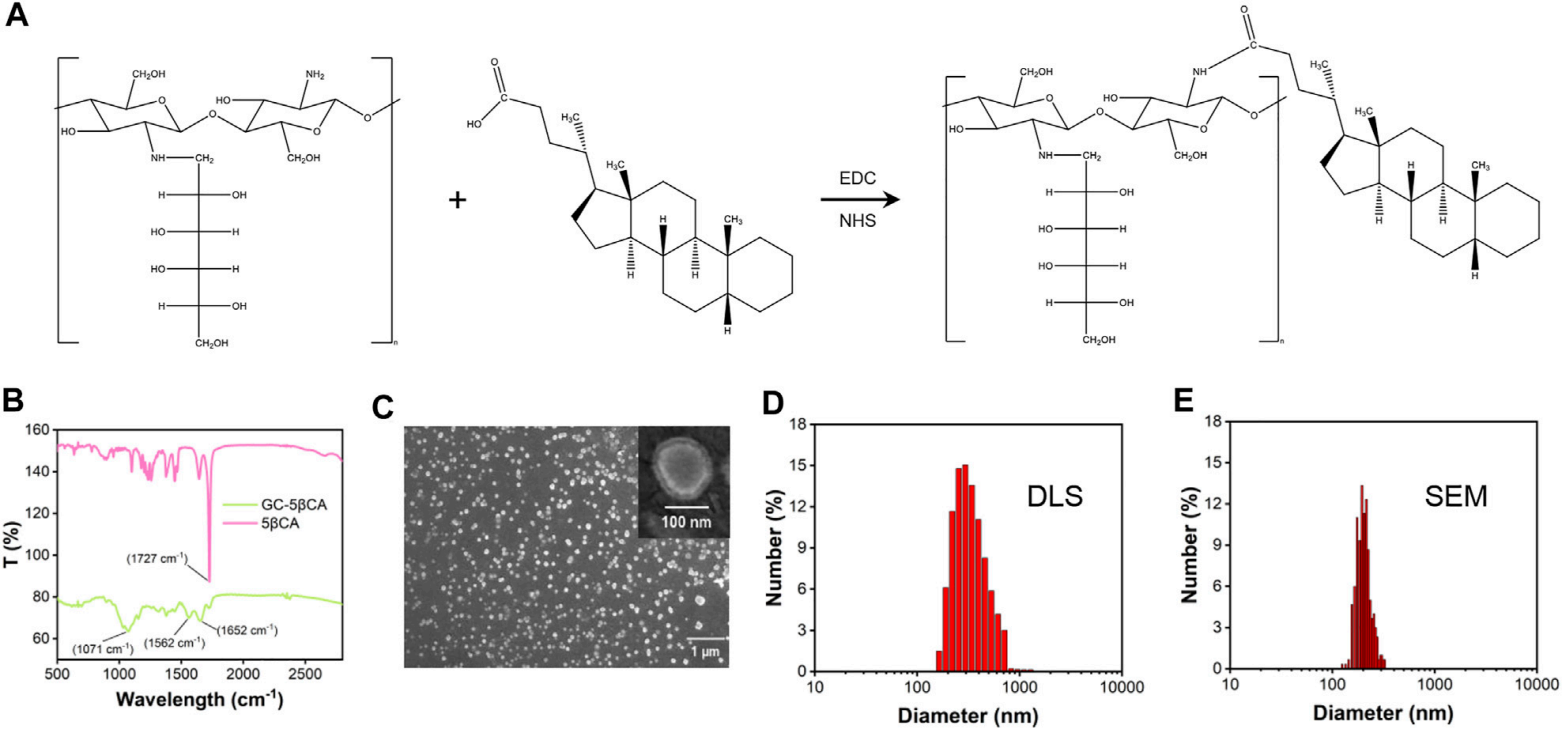
Characterization of GC@ICG. **(A)** Chemical structure of GC-5βCA conjugate. **(B)** The FTIR spectra of GC-5βCA and 5βCA. **(C)** SEM images of GC@ICG. Scale bar: 1 μm. Inset is enlarged image. Scale bar: 100 nm. **(D)** Size distribution of GC@ICG by DLS **(E)** Size distribution calculated from the SEM image.

**FIGURE 2:**
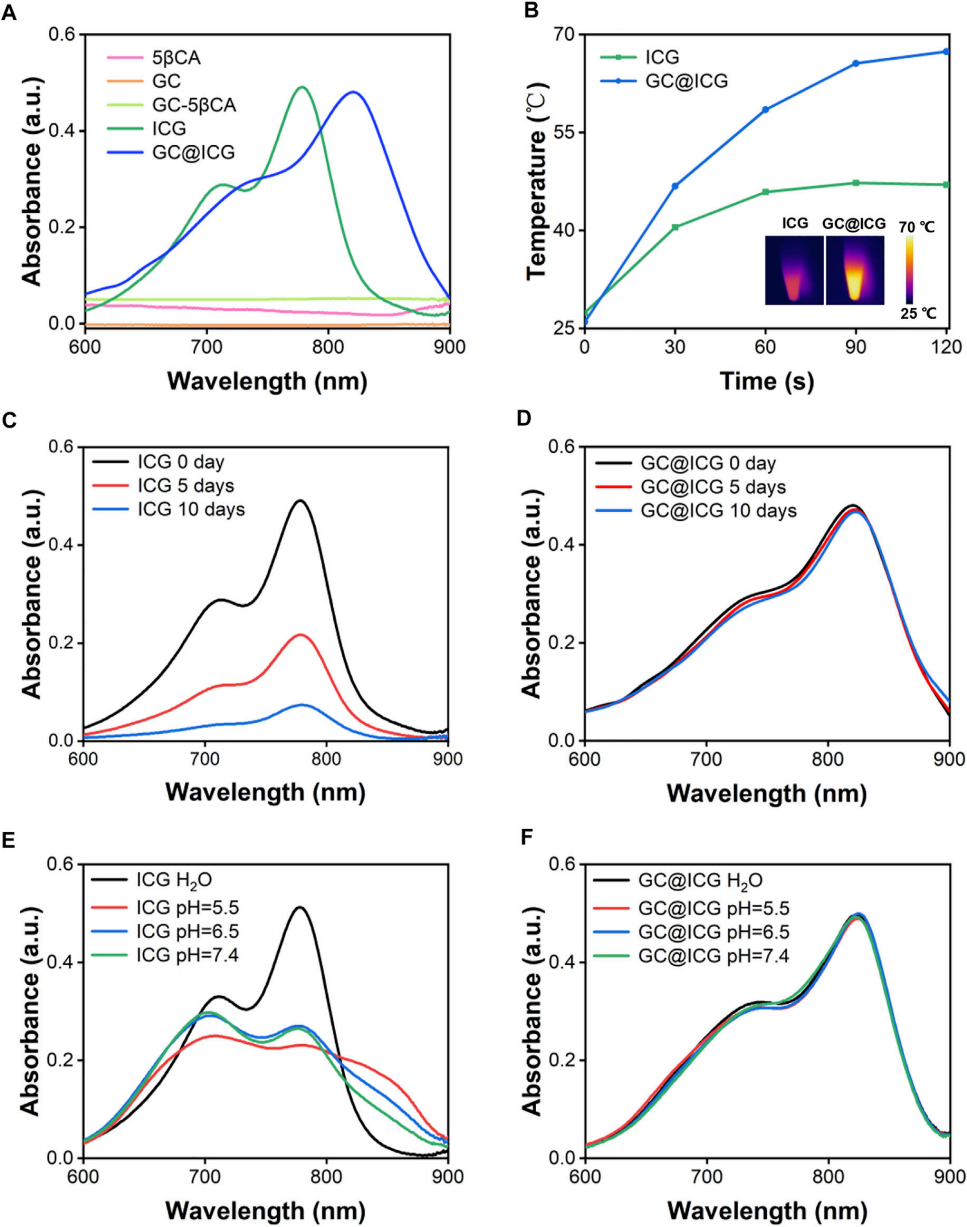
Absorption spectra of ICG and GC@ICG at different times and aqueous solutions. **(A)** UV-vis-NIR absorption spectra of ICG, GC@ICG, GC, 5βCA, and GC-5βCA. **(B)** Temperature curves of ICG and GC@ICG solution under 808 nm laser irradiation with a power density of 0.8 W/cm^2^ for 120 s. Inset: near-infrared thermal images of ICG and GC@ICG after laser irradiation for 120 s. Absorption spectra of ICG **(C)** and GC@ICG **(D)** at different times (0, 5, and 10 days). Absorption spectra of ICG **(E)** and GC@ICG **(F)** in different aqueous solutions (pH 5.5, 6.5, 7.4 and H_2_O).

GC@ICG particles were constructed *via* one step, assisted self-assembly procedures (Figure 1A). In order to enable GC with self-assembly functionality, 5βCA was introduced to be covalently attached to GC, thus producing polymeric amphiphiles, as mentioned in the previous report (Ryu et al., 2020). FTIR was adopted to prove the formation of GC-5βCA. As shown in Figure 1B, the absorption peak at 1727 cm^−1^ in the spectrum of 5βCA was attributed to the characteristic absorption of C=O stretching in carboxylic groups, and this absorption disappeared in GC-5βCA due to the formation of the amide bond. In the spectrum of GC-5βCA, the peaks that appeared at around 1,071 and 1,652 cm^−1^ were attributed to the characteristic absorptions of C-OH and N-H stretching, respectively. Particularly, the band at 1,562 cm^−1^ was attributed to amide absorption, indicating the successful binding of the 5βCA carboxylic acid group to the GC amino group through the amide bond (Girard et al., 2011; Lou et al., 2016; Fatouh et al., 2021). Upon contact with an aqueous environment, GC-5βCA spontaneously form nanoparticles with the encapsulation of ICG, named GC@ICG. Then, the morphology of GC@ICG was characterized by SEM. As revealed by the images, the GC@ICG showed spherical morphology with good dispersion and uniformity (Figure 1C). The hydrodynamic diameter of GC@ICG was larger than that measured by SEM (207.8 ± 11.2 nm), which may be attributed to the hydrophilicity and surface charge of GC (Figures 1D,E). Such size is conducive to cellular uptake and tumor accumulation (Foroozandeh and Aziz, 2018). Then, the optical property was studied. As shown in Figure 2A, GC@ICG solution had a high absorption peak of around 822 nm with a broad absorption shoulder. Compared to pure ICG, GC@ICG exhibited a redshift of about 40 nm in UV-visible spectroscopy. This could be attributed to the encapsulation of ICG molecules within GC@ICG nanoparticles, which remarkably affected their microenvironment. Then, the photothermal properties of GC@ICG were further explored by detecting the temperature change under the laser irradiation of 808 nm. The results in Figure 2B showed that, under the condition of the same concentration of ICG, GC@ICG could cause a more dramatic temperature increase than that of free ICG with the increase of laser irradiation time. GC@ICG reached 67.4°C in 120 s, while ICG could only reach a maximum of 46.8°C, which showed a more efficient photothermal conversion performance of GC@ICG. As a photosensitizer approved for medical application, ICG suffers from its drawbacks of aqueous instability and photodegradation, limiting its use *in vitro* and *in vivo* (Hu et al., 2019). We hope that these defects can be circumvented in the constructed GC@ICG. In order to determine the stability of GC@ICG, absorption spectra of the ICG and GC@ICG at different times and different pH conditions were measured. As shown in Figures 2C,D, the absorbance of ICG dropped 77.8% within 10 days, while almost no change in the absorption of GC@ICG was observed within the same time. In order to further determine the stability of GC@ICG in different microenvironments *in vivo*, three buffers representing the pH of different microenvironments were selected—pH 7.4 for normal tissue, pH 6.5 for tumor microenvironment, and pH 5.5 for lysosomes—and the absorption properties of ICG and GC@ICG were measured (Saftig and Klumperman, 2009; Qiu and Xia, 2020; Yang et al., 2021). The absorption of dissociative ICG changed greatly in different pHs (Figure 2E), while the absorption spectra of GC@ICG showed negligible change in any of the three buffers (Figure 2F). Together, these results suggested that the GC@ICG we constructed had good photothermal conversion performance and stability, which is necessary for treating the tumor *in vivo*.

### Cell Effects of GC@ICG Under NIR Laser Irradiation

**FIGURE 3:**
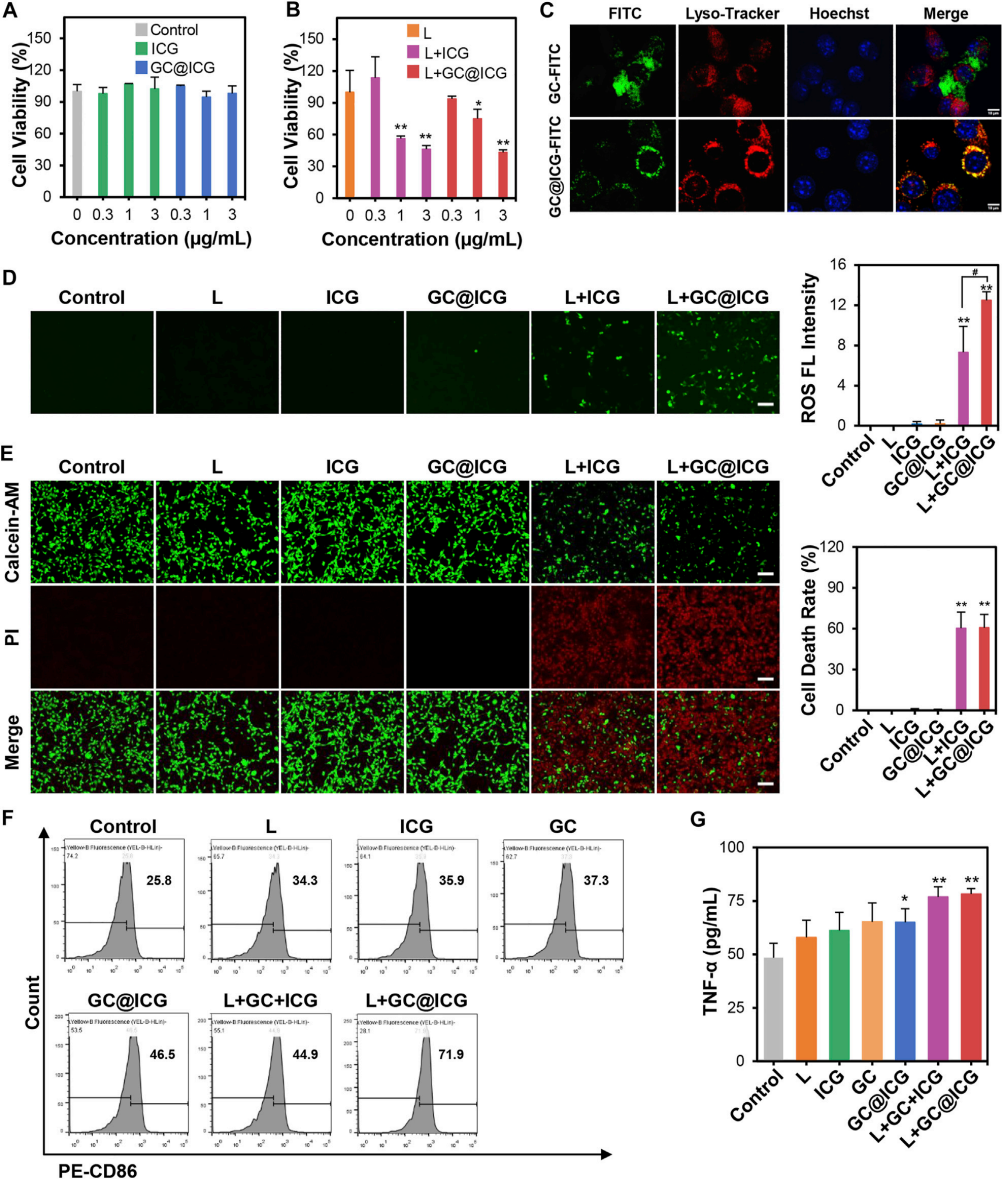
Immunogenic tumor cell death induced by GC@ICG NPs based phototherapy. Cell viability of 4T1 cells after incubation with different concentrations of ICG (0.3, 1, and 3 μg/ml) without **(A)** or with **(B)** 808 nm at 0.8 W/cm^2^ for 5 min (*n* = 3, *∗p* < 0.05, *∗∗p* < 0.01). **(C)** Fluorescence images of intracellular localization of GC and GC@ICG in 4T1 cells. Scale bar: 10 μm. **(D)** Fluorescence images of ROS production in 4T1 cells stained with DCFH-DA (a ROS probe, green). Scale bar: 100 μm. Bar graph demonstrated ROS fluorescence intensity (*n = 3, ∗∗p <* 0.01 and ^#^ *p <* 0.05 *vs.* indicated groups). **(E)** Fluorescence images of 4T1 cells stained with calcein-AM/PI for cell death evaluation after different treatments. Live cells: green, calcein-AM. Dead cells: red, PI. Scale bar: 100 μm. Bar graph demonstrated percentage of cell death rate (*n* = 3, *∗∗p* < 0.01). **(F)** Flow cytometric analysis of CD86 expression on the surface of DCs with different treatments. **(G)** ELISA analysis of TNF-α released from DCs stimulated by GC and GC@ICG (*n* = 3, *∗p* < 0.05, *∗∗p* < 0.01).

The cytotoxicity of GC@ICG under laser irradiation was determined by the CCK-8 method. 4T1 tumor cells were co-cultured with different concentrations of GC@ICG and irradiated with an 808-nm laser. The results indicated that GC@ICG showed almost no toxicity on 4T1 cells, indicating the high biocompatibility of GC@ICG. Meanwhile, the cell viability was 43.27% when co-cultured with GC@ICG (ICG 3 μg/ml) and 808 nm irradiation, indicating its photo-activated cytotoxicity (Figures 3A,B). Then, the capability of GC@ICG into cells was evaluated by FITC functionalized GC@ICG (GC@ICG-FITC) and incubating GC@ICG-FITC or GC-FITC with 4T1 cells in culture media, which were monitored by confocal laser scanning microscopy. Previous studies have shown that nanoparticles with a diameter of 200–300 nm could enter cells through different pathways and be trafficked to lysosomes (Behzadi et al., 2017; Vtyurina et al., 2021). Therefore, LysoTracker, a lysosome-specific red fluorescent probe, was used to judge the successful entry of GC@ICG into cells. According to the results of co-staining experiments, compared to GC-FITC, which can only slowly disperse extracellularly, co-localization between GC@ICG and LysoTracker was largely matched, exhibiting its ability to enter cells and that endocytosis was the pathway for the cellular internalization of GC@ICG (Figure 3C). The ROS-producing capacity of GC@ICG in 4T1 cells was evaluated by staining cells with DCFH-DA as the ROS probe. As shown in Figure 3D, cells treated with L + ICG and L + GC@ICG showed green fluorescence, indicating the generation of ROS. Particularly, the L + GC@ICG group exhibited significantly higher fluorescence intensity compared with the L + ICG group, showing that GC@ICG under laser irradiation could enhance endogenous oxidative stress than free ICG under laser irradiation. Then, the viability of 4T1 cells in different treatment groups was evaluated by calcein-AM/PI double staining, and the results demonstrated that most of the cells were stained with PI in the group of L + GC@ICG, indicating the killing effect of GC@ICG in cells under NIR light irradiation. In contrast, almost no dead cells were detected in other groups (Figure 3E). These results may be because the GC@ICG nanoparticles can enter cells directly, thus having a more effective killing effect on cells. It is reported that apoptotic tumor cells could stimulate the antitumor immune response (Feng et al., 2003; Ahmed and Tait, 2020). However, weak tumor cell immunogenicity is a barrier in immunotherapy. Using immunoadjuvants to enhance the specific immune response of the antigen is one of the breakthroughs to solve this problem. To our knowledge, GC is a potent immunoadjuvant used in laser immunotherapy to induce and enhance antitumor immune responses (Zhou F et al., 2018; Qi et al., 2020). Therefore, we further determined the immunological effects of GC@ICG on DCs by analyzing the upregulation of CD86, highly expressed on the surface of DCs, CD86 expression and cytokine release are typical markers of DC maturation. Flow cytometry was used to measure the upregulation of CD86 on DCs stimulated by treated tumor cells. As shown in Figure 3F, compared to the control group, tumor cells treated with L + GC@ICG could significantly promote the maturation of DCs. The expression of TNF-α was also upregulated in the group of L + GC@ICG (Figure 3G). Overall, these results indicated the photothermal effect of GC@ICG under NIR laser and the immune stimulation of the cells. Furthermore, the ability of GC@ICG to enter cells allowed it to be considered an “endogenous antigen” to be recognized by antigen presenting cells when a tumor cell dies, thereby enhancing tumor cell destruction and antitumor immune response.

### *In Vivo* Effect of GC@ICG-Based Phototherapy

**FIGURE 4:**
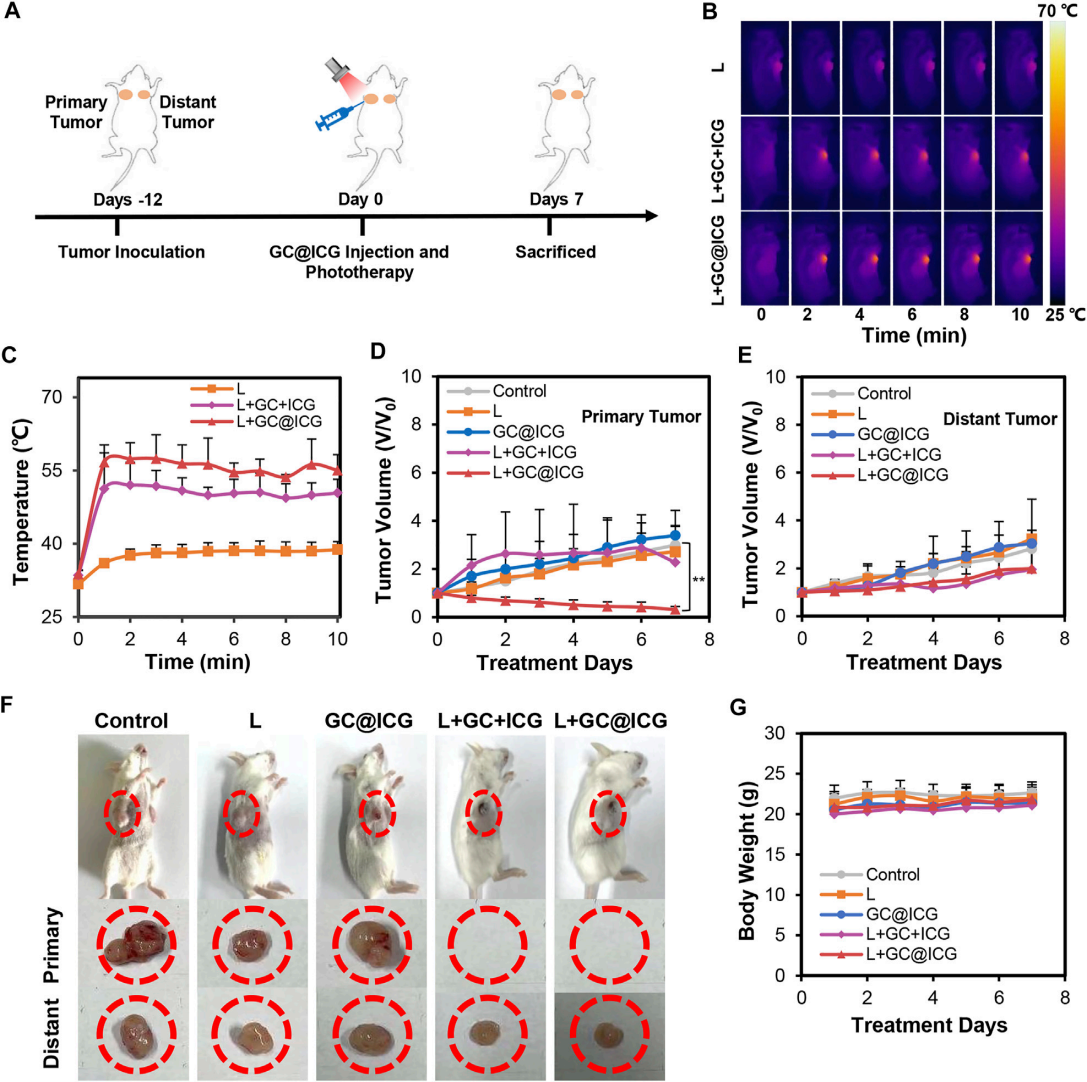
*In vivo* antitumor effect of GC@ICG-based phototherapy. **(A)** Schematic illustration of the timeline of the animal experiment for GC@ICG-based phototherapy. **(B)** Near-infrared thermal images of mice during 808 nm laser irradiation at 0.8 W/cm^2^ for 10 min. **(C)** Temperature elevations on primary tumor surface during laser irradiation based on infrared thermal imaging. Time-dependent tumor growth curves of primary **(D)** (*n* = 3, *∗∗p* < 0.01) and distant tumors **(E)**. **(F)** Photographs of mice with different treatments (from left to right: control; L; GC@ICG; L + GC + ICG; L + GC@ICG). **(G)** The change of body weights of mice after different treatments.

**FIGURE 5:**
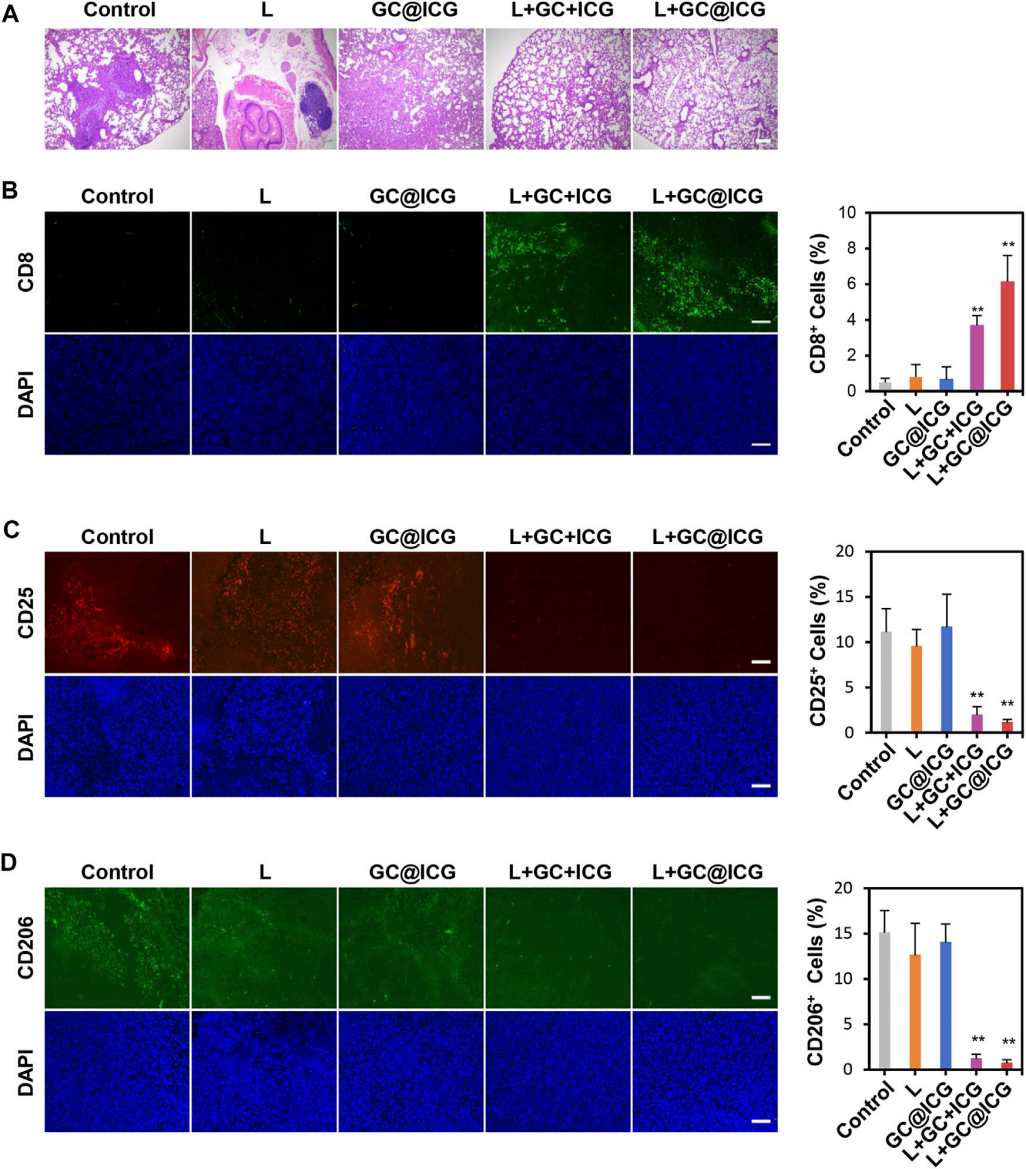
Abscopal effect of GC@ICG-based phototherapy. **(A)** H&E staining images of lungs collected from mice after different treatments. Scale bar: 200 μm. Immunofluorescence images of the excised distant tumor stained with anti-CD8a **(B)**, anti-CD25 **(C)**, and anti-CD206 **(D)**. Bar graphs showed the number of CD8^+^, CD25^+^, and CD206^+^ cells infiltrated in distant tumors. Scale bar: 100 μm (*n* = 3, *∗∗p* < 0.01).

Having proved the killing effect and immune promotion effect of GC@ICG on tumor cells at the cellular level, we next sought to study the *in vivo* PIT treatment efficacy of GC@ICG in mice. According to the treatment schedule illustrated in Figure 4A, the therapeutic effect of GC@ICG-based phototherapy was evaluated. First, mice bearing 4T1 breast tumors were established by injecting tumor cells into the breast of both sides of BALB/C female mice. Then, the mice were randomly assigned into five different groups, including control, L, GC@ICG, L + GC + ICG, and L + GC@ICG groups (*n* = 3). The temperature of the tumor surface under the laser irradiation with an 808-nm laser of 0.8 W/cm^2^ for 10 min was recorded by an infrared thermal imaging camera. The results suggested that the L + GC@ICG group had a higher temperature rise rate and reached the maximum temperature of 56.7°C within 1 min. However, the surface temperature of mice in the group of L and L + GC + ICG only increased to 36°C and 51.2°C, respectively, demonstrating the excellent photothermal effect of GC@ICG *in vivo* (Figures 4B,C). Tumor volumes and body weights of the mice were recorded daily during the treatment, and all tested mice were sacrificed after 7 days of treatment. It was observed that GC@ICG could apparently inhibit the development of 4T1 tumors under laser irradiation on both sides and the primary tumor eventually disappeared completely after treatment. Additionally, the primary tumors of the mice in the L + GC + ICG group were also completely ablated, while the scabs at the tumor sites affected the measured value. However, mice treated with laser irradiation alone exhibited only a slight inhibition effect on tumors, and mice treated with GC@ICG alone showed a similar tumor growth rate to that of untreated mice, without any observed inhibition effect (Figures 4D–F). As shown in Figure 4G, neither death nor significant weight change in mice was observed within 7 days after treatment, indicating that our treatment strategy had excellent biosafety. Hematoxylin and eosin (H&E) staining of lung tissue slices demonstrated that the strongest inhibition effect of metastasis was obtained with the combination therapy of GC@ICG and 808 nm laser irradiation (Figure 5A). Meanwhile, the CD8 immunohistochemical staining of distant tumor slices showed that the L + GC + ICG and L + GC@ICG groups exhibited the infiltration of CD8^+^ T cells, while the expression of CD8 in the L + GC@ICG group was significantly higher than that in the L + GC + ICG group, which proved that compared with the simple mixture of ICG and GC, the corresponding stimulation of GC@ICG to the immune system was obviously stronger (Figure 5B). Meanwhile, regulatory T cells (Tregs, marked by CD25) and M2 macrophage (marked by CD206) significantly decreased in distant tumors after L + GC@ICG treatment, indicating the decreased immunosuppression in the tumor microenvironment (Figures 5C,D). This further demonstrated the great advantages of GC@ICG nanoparticles over other GC-based systems in terms of photo-immunotherapy.

## Conclusion

In summary, we developed a self-assembled nanoparticle GC@ICG for photo-immunotherapy of TNBC. The self-assembled GC@ICG nanoparticles can act as “Trojan Horses” to deliver GC into tumor cells efficiently. Laser-induced temperature rise can cause cell death and release DAMPs, antigens, and GC@ICG. When DC recognizes DAMPs, GC can further stimulate DC as an immune stimulator and enhance the host antitumor immune response. Inhibited primary tumors and distant tumors were achieved by taking advantage of the above-mentioned excellent properties of GC@ICG, indicating an extraordinary therapeutic effect. Therefore, the oncotherapy strategy we constructed is a promising candidate for tumor treatment, which will provide a new approach for clinical application in cancer diagnosis and treatment in the future.

## Data Availability

The original contributions presented in the study are included in the article further inquiries can be directed to the corresponding authors.

## References

[B1] Ahmed A. Tait S. W. G. (2020). Targeting Immunogenic Cell Death in Cancer. Mol. Oncol. 14, 2994–3006. 10.1002/1878-0261.12851 33179413 PMC7718954

[B2] Behzadi S. Serpooshan V. Tao W. Hamaly M. A. Alkawareek M. Y. Dreaden E. C. (2017). Cellular Uptake of Nanoparticles: Journey inside the Cell. Chem. Soc. Rev. 46, 4218–4244. 10.1039/c6cs00636a 28585944 PMC5593313

[B3] Chen R. Wang X. Yao X. Zheng X. Wang J. Jiang X. (2013). Near-IR-Triggered Photothermal/Photodynamic Dual-Modality Therapy System via Chitosan Hybrid Nanospheres. Biomaterials 34, 8314–8322. 10.1016/j.biomaterials.2013.07.034 23896004

[B4] Chen Q. Xu L. Liang C. Wang C. Peng R. Liu Z. (2016). Photothermal Therapy with Immune-Adjuvant Nanoparticles Together with Checkpoint Blockade for Effective Cancer Immunotherapy. Nat. Commun. 7, 13193. 10.1038/ncomms13193 27767031 PMC5078754

[B5] Fatouh A. M. Elshafeey A. H. Abdelbary A. (2021). Galactosylated Chitosan Coated Liposomes of Ledipasvir for Liver Targeting: Chemical Synthesis, Statistical Optimization, *In-Vitro* and *In-Vivo* Evaluation. J. Pharm. Sci. 110, 1148–1159. 10.1016/j.xphs.2020.10.002 33039437

[B6] Feng H. Zeng Y. Graner M. W. Likhacheva A. Katsanis E. (2003). Exogenous Stress Proteins Enhance the Immunogenicity of Apoptotic Tumor Cells and Stimulate Antitumor Immunity. Blood 101, 245–252. 10.1182/blood-2002-05-1580 12393411

[B41] Foroozandeh P. Aziz A. A. (2018). Insight into Cellular Uptake and Intracellular Trafficking of Nanoparticles. Nanoscale Res Lett. 13, 1–12. 10.1186/s11671-018-2728-6 30361809 PMC6202307

[B7] Garrido-Castro A. C. Lin N. U. Polyak K. (2019). Insights into Molecular Classifications of Triple-Negative Breast Cancer: Improving Patient Selection for Treatment. Cancer Discov. 9, 176–198. 10.1158/2159-8290.CD-18-1177 30679171 PMC6387871

[B8] Girard H. A. Petit T. Perruchas S. Gacoin T. Gesset C. Arnault J. C. (2011). Surface Properties of Hydrogenated Nanodiamonds: A Chemical Investigation. Phys. Chem. Chem. Phys. 13, 11517–11523. 10.1039/c1cp20424f 21566816

[B9] Gordon S. R. Maute R. L. Dulken B. W. Hutter G. George B. M. McCracken M. N. (2017). PD-1 Expression by Tumour-Associated Macrophages Inhibits Phagocytosis and Tumour Immunity. Nature 545, 495–499. 10.1038/nature22396 28514441 PMC5931375

[B10] Hu H. Chen J. Yang H. Huang X. Wu H. Wu Y. (2019). Potentiating Photodynamic Therapy of ICG-Loaded Nanoparticles by Depleting GSH with PEITC. Nanoscale 11, 6384–6393. 10.1039/c9nr01306g 30888375

[B11] Hu D. Xu H. Zhang W. Xu X. Xiao B. Shi X. (2021). Vanadyl Nanocomplexes Enhance Photothermia-Induced Cancer Immunotherapy to Inhibit Tumor Metastasis and Recurrence. Biomaterials 277, 121130. 10.1016/j.biomaterials.2021.121130 34534862

[B12] Huang X. Lu Y. Guo M. Du S. Han N. (2021). Recent Strategies for Nano-Based PTT Combined with Immunotherapy: From a Biomaterial Point of View. Theranostics 11, 7546–7569. 10.7150/thno.56482 34158866 PMC8210617

[B13] Hucks G. Rheingold S. R. (2019). The Journey to CAR T Cell Therapy: The Pediatric and Young Adult Experience with Relapsed or Refractory B-ALL. Blood Cancer J. 9, 10–19. 10.1038/s41408-018-0164-6 30670684 PMC6342933

[B14] Hwang S. Y. Park S. Kwon Y. (2019). Recent Therapeutic Trends and Promising Targets in Triple Negative Breast Cancer. Pharmacol. Ther. 199, 30–57. 10.1016/j.pharmthera.2019.02.006 30825473

[B15] Li X. Ferrel G. L. Guerra M. C. Hode T. Lunn J. A. Adalsteinsson O. (2011). Preliminary Safety and Efficacy Results of Laser Immunotherapy for the Treatment of Metastatic Breast Cancer Patients. Photochem. Photobiol. Sci. 10, 817–821. 10.1039/c0pp00306a 21373701 PMC5976443

[B16] Liu X. Zheng C. Kong Y. Wang H. Wang L. (2022). An *In Situ* Nanoparticle Recombinant Strategy for the Enhancement of Photothermal Therapy. Chin. Chem. Lett. 33, 328–333. 10.1016/j.cclet.2021.07.025

[B17] Lou R. Xie H. Zheng H. Ren Y. Gao M. Guo X. (2016). Alginate-Based Microcapsules with Galactosylated Chitosan Internal for Primary Hepatocyte Applications. Int. J. Biol. Macromol. 93, 1133–1140. 10.1016/j.ijbiomac.2016.09.078 27667543

[B18] Nam J. Son S. Ochyl L. J. Kuai R. Schwendeman A. Moon J. J. (2018). Chemo-Photothermal Therapy Combination Elicits Anti-tumor Immunity against Advanced Metastatic Cancer. Nat. Commun. 9, 1074. 10.1038/s41467-018-03473-9 29540781 PMC5852008

[B19] Ng C. W. Li J. Pu K. (2018). Recent Progresses in Phototherapy-Synergized Cancer Immunotherapy. Adv. Funct. Mater. 28, 1804688. 10.1002/adfm.201804688

[B20] Poggio F. Bruzzone M. Ceppi M. Pondé N. F. La Valle G. Del Mastro L. (2018). Platinum-Based Neoadjuvant Chemotherapy in Triple-Negative Breast Cancer: A Systematic Review and Meta-Analysis. Ann. Oncol. 29, 1497–1508. 10.1093/annonc/mdy127 29873695

[B21] Qi S. Lu L. Zhou F. Chen Y. Xu M. Chen L. (2020). Neutrophil Infiltration and Whole-Cell Vaccine Elicited by N-Dihydrogalactochitosan Combined with NIR Phototherapy to Enhance Antitumor Immune Response and T Cell Immune Memory. Theranostics 10, 1814–1832. 10.7150/thno.38515 32042338 PMC6993227

[B22] Qiu J. Xia Y. (2020). Killing Cancer Cells by Rupturing Their Lysosomes. Nat. Nanotechnol. 15, 252–253. 10.1038/s41565-020-0639-z 32203434

[B23] Ryu J. H. Yoon H. Y. Sun I. C. Kwon I. C. Kim K. (2020). Tumor-Targeting Glycol Chitosan Nanoparticles for Cancer Heterogeneity. Adv. Mater. 32, e2002197. 10.1002/adma.202002197 33051905

[B24] Saftig P. Klumperman J. (2009). Lysosome Biogenesis and Lysosomal Membrane Proteins: Trafficking Meets Function. Nat. Rev. Mol. Cel Biol. 10, 623–635. 10.1038/nrm2745 19672277

[B25] Sato K. Ando K. Okuyama S. Moriguchi S. Ogura T. Totoki S. (2018). Photoinduced Ligand Release from a Silicon Phthalocyanine Dye Conjugated with Monoclonal Antibodies: A Mechanism of Cancer Cell Cytotoxicity after Near-Infrared Photoimmunotherapy. ACS Cent. Sci. 4, 1559–1569. 10.1021/acscentsci.8b00565 30555909 PMC6276043

[B26] Siegel R. L. Miller K. D. Fuchs H. E. Jemal A. (2021). Cancer Statistics, 2021. CA Cancer J. Clin. 71, 7–33. 10.3322/caac.21654 33433946

[B27] Sung H. Ferlay J. Siegel R. L. Laversanne M. Soerjomataram I. Jemal A. (2021). Global Cancer Statistics 2020: GLOBOCAN Estimates of Incidence and Mortality Worldwide for 36 Cancers in 185 Countries. CA Cancer J. Clin. 71, 209–249. 10.3322/caac.21660 33538338

[B28] van Puffelen J. H. Keating S. T. Oosterwijk E. van der Heijden A. G. Netea M. G. Joosten L. A. B. (2020). Trained Immunity as a Molecular Mechanism for BCG Immunotherapy in Bladder Cancer. Nat. Rev. Urol. 17, 513–525. 10.1038/s41585-020-0346-4 32678343

[B29] Vtyurina N. Åberg C. Salvati A. (2021). Imaging of Nanoparticle Uptake and Kinetics of Intracellular Trafficking in Individual Cells. Nanoscale 13, 10436–10446. 10.1039/d1nr00901j 34076024 PMC8211015

[B30] Waks A. G. Winer E. P. (2019). Breast Cancer Treatment: A Review. JAMA 321, 288–300. 10.1001/jama.2018.19323 30667505

[B31] Wang H. Li X. Tse B. W. Yang H. Thorling C. A. Liu Y. (2018). Indocyanine Green-Incorporating Nanoparticles for Cancer Theranostics. Theranostics 8, 1227–1242. 10.7150/thno.22872 29507616 PMC5835932

[B32] Wang M. Song J. Zhou F. Hoover A. R. Murray C. Zhou B. (2019). NIR-Triggered Phototherapy and Immunotherapy via an Antigen-Capturing Nanoplatform for Metastatic Cancer Treatment. Adv. Sci. (Weinh) 6, 1802157. 10.1002/advs.201802157 31131193 PMC6523374

[B33] Wang H. Najibi A. J. Sobral M. C. Seo B. R. Lee J. Y. Wu D. (2020). Biomaterial-Based Scaffold for *In Situ* Chemo-Immunotherapy to Treat Poorly Immunogenic Tumors. Nat. Commun. 11, 5696. 10.1038/s41467-020-19540-z 33173046 PMC7655953

[B34] Wang M. Rao J. Wang M. Li X. Liu K. Naylor M. F. (2021). Cancer Photo-Immunotherapy: From Bench to Bedside. Theranostics 11, 2218–2231. 10.7150/thno.53056 33500721 PMC7797676

[B35] Weiss S. A. Wolchok J. D. Sznol M. (2019). Immunotherapy of Melanoma: Facts and Hopes. Clin. Cancer Res. 25, 5191–5201. 10.1158/1078-0432.CCR-18-1550 30923036 PMC6726509

[B36] Yang Z. Li P. Chen Y. Gan Q. Feng Z. Jin Y. (2021). Construction of pH/Glutathione Responsive Chitosan Nanoparticles by a Self-Assembly/Self-Crosslinking Method for Photodynamic Therapy. Int. J. Biol. Macromol. 167, 46–58. 10.1016/j.ijbiomac.2020.11.141 33271181

[B37] Yin L. Duan J. J. Bian X. W. Yu S. C. (2020). Triple Negative Breast Cancer Molecular Subtyping and Treatment Progress. Breast Cancer Res. 22, 61. 10.1186/s13058-020-01296-5 32517735 PMC7285581

[B38] Zhou B. Song J. Wang M. Wang X. Wang J. Howard E. W. (2018). BSA-bioinspired Gold Nanorods Loaded with Immunoadjuvant for the Treatment of Melanoma by Combined Photothermal Therapy and Immunotherapy. Nanoscale 10, 21640–21647. 10.1039/c8nr05323e 30232481 PMC6265078

[B39] Zhou F. Wu S. Song S. Chen W. R. Resasco D. E. Xing D. (2012). Antitumor Immunologically Modified Carbon Nanotubes for Photothermal Therapy. Biomaterials 33, 3235–3242. 10.1016/j.biomaterials.2011.12.029 22296829 PMC5987227

[B40] Zhou F. Yang J. Zhang Y. Liu M. Lang M. L. Li M. (2018). Local Phototherapy Synergizes with Immunoadjuvant for Treatment of Pancreatic Cancer through Induced Immunogenic Tumor Vaccine. Clin. Cancer Res. 24, 5335–5346. 10.1158/1078-0432.CCR-18-1126 30068705 PMC6214772

